# Conformal Cooling Channel Design for Improving Temperature Distribution on the Cavity Surface in the Injection Molding Process

**DOI:** 10.3390/polym15132793

**Published:** 2023-06-23

**Authors:** Van-Thuc Nguyen, Pham Son Minh, Tran Minh The Uyen, Thanh Trung Do, Nguyen Canh Ha, Van Thanh Tien Nguyen

**Affiliations:** 1 Faculty of Mechanical Engineering, Ho Chi Minh City University of Technology and Education, Ho Chi Minh City 71307, Vietnam; 2 Faculty of Mechanical Engineering, Industrial University of Ho Chi Minh City, Nguyen Van Bao Street, Ward 4, Go Vap District, Ho Chi Minh City 70000, Vietnam

**Keywords:** plastic injection molding, cooling channel, mold heating, response surface methodology

## Abstract

Mold heating is an essential process in plastic injection molding. Raising the temperature of the mold before injecting liquefied plastic can ease the mold-filling process. A cooling channel can be used to transport high-temperature fluids for this purpose, such as hot water or oil. This dual purpose is a cost-effective solution for heating the mold because the target temperature is easily achieved using this method. In addition, a conformal cooling channel (CCC) can provide more efficient mold heating than a straight cooling channel. This study used the response surface methodology to determine the optimum CCC shape for heat distribution in a mold, and the simulation results confirmed its optimization. The average temperature of the mold using a CCC was better than that using a straight cooling channel, and the heat zone was uniform across the mold surface.

## 1. Introduction

Recently, additive manufacturing (AM) has emerged as a promising manufacturing method that, in many cases, could replace traditional manufacturing methods [1,2,3]. The original material shapes for the AM method are powder or wire, and they are melted and adhered to generate the designed forms. Powder materials require a laser beam or electron beam, which are expensive and high-energy consumption devices, despite the fact that they could build high-resolution parts [4,5,6,7,8]. Compared to powder materials, wire shapes have the merits of saving fusion energy and time during the additive process. Significantly, the metal wire could create high mechanical characteristics that could be applied in marine, aerospace, and automobile industries [9,10,11,12].

The injection-molding process has been widely used to manufacture plastic products of various shapes and sizes owing to its high manufacturing productivity and low associated costs. This process can be separated into four steps: filling/injection, packing, cooling, and ejection. The cooling step is the most important of these four processes and constitutes the most extensive injection-cycle process. Contrastingly, this step severely affects the mold temperature, directly impacting shrinkage; S.-C. Nian et al. [1] showed that the optimal cooling channel system provides a better heat-transfer area. The heat quickly dissipated from the mold and the part deformation decreased by 37% in the warpage, welding line, and sink mark. As discussed by B. Ravikiran et al. [2], the cooling step is the most significant control factor, with a 29.04% contribution that affects the weld line width and sink mark depth. Moreover, these two factors affect the part strength and the optimal cooling time, which could reduce the weld line width and lead to 56.4% and 68.9% sink mark depth and filling ability, respectively, when melting into the cavity. During the cooling process, cooling fluid is used to lower the mold temperature through the cooling channel system, reducing the product temperature to induce solidification [3]. As cooling quality directly affects the quality of the product, temperature uniformity can restrict many defects, specifically, those associated with material warpage and shrinkage.

To prevent the injection issue as mentioned above, the mold temperature is the important factor. Many methods could be used to raise the mold temperature so the melted plastic could easily fill out the mold and prevent the injection issue. The effect of mold temperature on the filling ability of melted plastic has been studied. By using external induction to raise the mold temperature, the filling percentage of the product increased from 10.2% to 100% when heating the mold with the 5 mm inductor coil in 5 s [4]. The gas-assisted method also controls mold temperature, which shows an impressive effect on the injection process, especially with the thin product. With the gas-assisted method, mold temperature reached 158.4 °C in 20 s and the flow length increased from 37.85~41.32 mm with PP plastic and 14.54~15.8 mm with ABS plastic [6]. Hot gas heating could also improve the filling ratio on the microgroove by up to 91% [7]. This method also showed the effectiveness of the thin-wall product; with 300 °C hot gas assisted, the melted plastic could fill the cavity with a thickness of 0.5 mm and an area of 39 × 120 mm [8]. The above methods show high effectiveness of the injection process, but the disadvantage of that method is costly because they require an external component to raise the mold temperature. So, the cooling channel system could be used as an effective method. The cooling channel system is not only responsible for mold cooling through cooling-fluid distribution, but in many cases, it is also used as a straightforward and effective method to heat the mold. However, the shape and drilling process of straight-drilled channels limit their ability to reach optimal cooling and heating targets. The need to avoid cavity interference and the restriction to straight drilling inherently constrains the design of these channels, potentially compromising their cooling efficiency. A practical example of these limitations is evident when straight-drilled channels are utilized to cool a curved cavity. In such a case, the gap between the straight channel and the curved cavity varies, leading to uneven cooling rates across the cavity surface. This inconsistency in cooling can trigger temperature irregularities, culminating in variable shrinkage and potential warpage of the manufactured component [9,10,11].

Adding to these concerns, the ejection of the part from the mold can only happen when the entire cavity has cooled to below the ejection temperature. Consequently, cooling time hinges on both the highest temperature point within the cavity and its local cooling rate. In general, traditional cooling channel systems (straight cooling channels) are shown to be effective with simple product molds of uniform thickness, but in the case of complex product molds with varying thicknesses, conformal cooling channels (CCCs) are considered as they better conform to the mold surface.

Recently, there have been many studies on CCCs, focusing on their effectiveness during the cooling process and the impact this has on product quality. Shaiful et al. [12] analyzed material warpage using Autodesk mold flow and analyzed results using ANOVA and Taguchi. Factors such as the coolant temperature, melt temperature, packing pressure, and packing time affected the injection temperature. The results of these changes were compared to the warpage percent between the straight and CCCs. The results showed that the CCC produced less material warping with optimal parameters based on the cooling channel type than the straight cooling channel (0.2826 mm versus 0.3005 mm). Vojnová et al. [13] studied the benefits of the CCC system in the molding process. The spiral cooling channel was developed, and analysis shows that cooling and production cycle times reduced up to 20% and 10%, respectively. Li et al. [14] studied topology optimization to design CCCs. This study uses the boundary-element method (BEM) for optimizing the cooling channel. An optimal geometric and topologic structure can be obtained for the cooling system by deleting invalid channel sections. The result showed that the cooling effectiveness increased by more than 50% with each case study. However, this has limited practical application as the resulting cooling channel is difficult to manufacture using a traditional machining method.

To address the challenge of manufacturing conformal cooling channels, several solutions [15,16,17] have been suggested. Because of the intricate 3D internal configurations that are a hallmark of optimal conformal cooling (CC) designs, traditional mechanical cutting techniques (subtractive manufacturing) cannot be employed to machine CC channels. Several methodologies for fabricating CC molds have been suggested since the late 1990s, each with its own constraints:

- Casting [18,19,20]: While casting can produce complex structures, the accuracy and resolution might be compromised, especially for intricate designs. It is also labor-intensive and may not be suitable for high volume production.
- Milled groove method [21,22,23,24,25]: This method provides better accuracy compared to casting, but it is not feasible for extremely complex geometries. The longevity of the mold might also be compromised due to the stress concentration in the grooved area.
- Laminated tooling [26,27,28,29,30]: While this method can handle more complex designs, the alignment of layers might be challenging, potentially impacting the accuracy and quality of the final mold.
- Powder-based additive manufacturing [31,32,33,34]: This method offers great freedom of design, but the resulting molds often have a rough surface finish. It can also be time-consuming and costly, especially for large parts.
- Hybrid additive/subtractive manufacturing [35,36,37]: This combines the best of both worlds but can be complex to implement and control. It may also require specialized equipment, which increases the overall cost.

To address the aforementioned limitations in existing techniques, this research proposes a new method that combines the wire arc additive manufacturing (WAAM) method with conventional milling [38]. This innovative approach aims to shape the mold plate to include the conformal cooling channel. The following highlights detail the myriad of advantages of this combination:

- Unleashing creative design possibilities: WAAM facilitates the construction of intricate geometries that traditional methods find challenging or impossible. Once these complex structures are erected, milling steps in to refine and perfect the part. This collaborative effort results in delivering precision and enhancing the quality of the meticulously designed shapes.
- Material and cost efficiency: WAAM, a form of directed energy deposition (DED) technology, provides stellar material usage efficiency. Since it primarily engages the deposition of wire-fed materials, the resultant waste is negligible, leading to a significant reduction in raw material costs. Further, the subtractive nature of milling is carefully regulated to cut down waste, fostering even greater cost-efficiency.
- Time-efficient production: The impressive deposition rates of WAAM facilitate a swift production of large components or molds. Subsequently, milling refines these products rapidly and accurately. This amalgamation of processes potentially leads to substantial time savings, especially when compared to the utilization of either technique in isolation.
- Enhancement of mechanical properties: The layer-by-layer additive procedure inherent to WAAM can culminate in the refinement of mechanical properties, brought about by rapid cooling and grain refinement. This enhancement can be especially observable in the case of alloyed materials.
- Repair and remanufacturing capabilities: The synergy of WAAM and milling also proves beneficial for mold repair or remanufacturing. It adds material precisely where required and mills it to achieve the perfect, final shape. This strategy prolongs the lifecycle of molds or parts, eliminating the need for a complete overhaul.
- Hybrid flexibility: The integration of both additive and subtractive methods within a single setup paves the way for remarkable flexibility. By transitioning between processes as required, manufacturers can fine-tune different aspects of production, such as material usage, surface finish, and dimensional accuracy.

While this method brings about several advantages, it is also critical to consider potential challenges such as managing heat input to prevent warping or distortion, overseeing the transition between additive and subtractive processes, and ensuring the compatibility of equipment.

As a solution, additive manufacturing, such as metal three-dimension printing, could create this type of cooling channel. Park and Dang [39] have developed an injection mold with a CCC. They tried to solve an automotive part’s uneven cooling and long cycle times of the molding process. The result shows that the CCC has a more uniform temperature distribution than the traditional cooling channel, and the cooling time is reduced by more than 30%. In 2017, Suchanan et al. [40] investigated the optimization of CCCs by analyzing the cooling time and thermal-mechanical mold interactions. The authors used designs based on experimentation to research the effect of critical design parameters of CCCs on the cooling time and thermo-mechanical performance of molds. However, this research is still limited to simulation; the actual results of this cooling channel still need to be verified by experimentation. Venkatesh and Kumar [41] also analyzed the thermal performance of CCCs. This simulation changed the cooling shape (circle, trapezoid, and rectangle), path (straight, spiral, and spiral square), and cooling distance to find the optimum cooling shape. The results show that a spiral path CCC with a circle profile achieves the minimum surface temperature. A CCC shows a better temperature distribution in the cooling process than a straight cooling channel (90.36 °C versus 100.33 °C).

As previously mentioned, most of the research on CCCs has focused on analyzing the cooling process. However, it is essential to note that mold heating plays a critical role in the injection process. It has been found that higher mold temperatures result in better flow characteristics for melting plastic and filling the mold. In this study, we have used a CCC in the heating process and optimized it by carefully considering the temperature distributions of the mold at the end of both the heating and cooling steps. We utilized the response surface methodology to optimize the cooling channels to achieve this optimization.

## 2. Simulation and Experimental Methods

### 2.1. Design Products for Experiments

**Figure 1:**
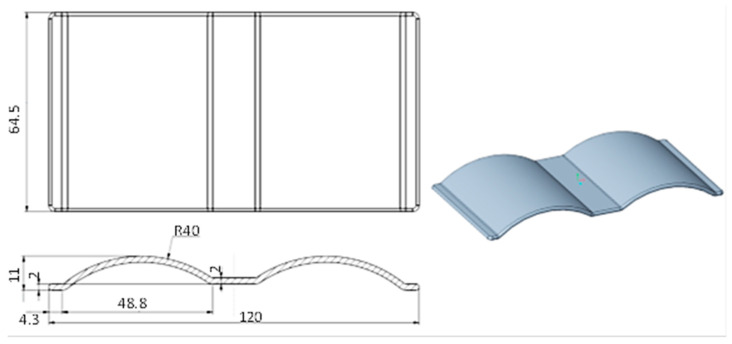
Detailed drawing of the experimental product.

In previous studies on mold-temperature control for injection molding [42,43,44,45,46,47], the straight cooling channel was used with different heating methods. However, more realistic products will have many complex cavity-surface geometries. Therefore, the distance between the cooling channel and the cavity surface will vary. This issue will lead to different heat transfer rates from the hot-melt plastic to the wall of the cooling channel, resulting in an imbalance of temperature at the cavity surface. Thus, to mitigate this problem, the CCC was suggested for balancing the heat transfer from the cavity surface to the cooling channel, which will lead to improved temperature distribution on the cavity wall surface and machine part temperatures. The products in Figure 1 with a plate and wave profile were chosen for the experiment in a previous study concerned with optimizing the cooling channel shape. The wave profile resulted in a non-uniform mold thickness; therefore, using a straight cooling channel will result in unbalanced temperature distributions for this product geometry.

### 2.2. Mold Design

In this study, we aimed to optimize the cooling geometry to improve the temperature distribution on the mold face. Thus, only the mold plate was designed, simulated, and subjected to experimentation. We selected one product in the cavity and two cooling channels. The cooling channel design should consider the thickness of the experimental product; therefore, the channel diameter is based on the product thickness. If the channel diameter is d (mm), the distance between the centerline of the two channels should be between 3d and 4d, and the distance from the channel centerline to the mold surface should be between 2d and 3d. Guidelines for the cooling parameters are shown in Table 1.

**Figure 2:**
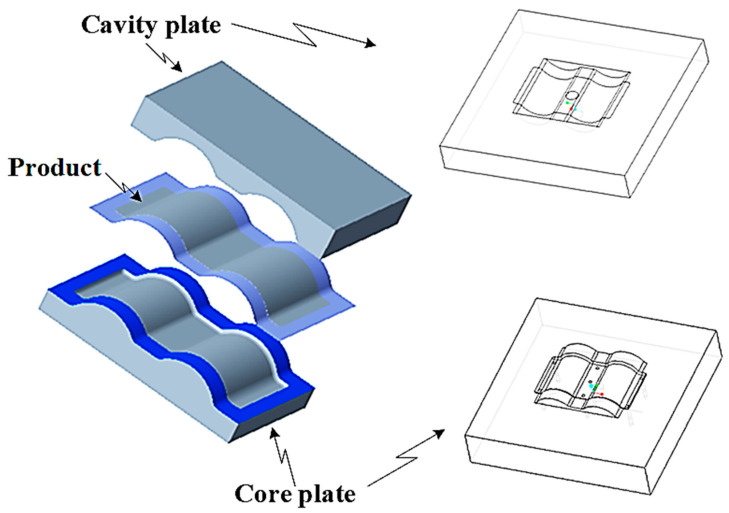
CAD model of the injection mold.

**Figure 3:**
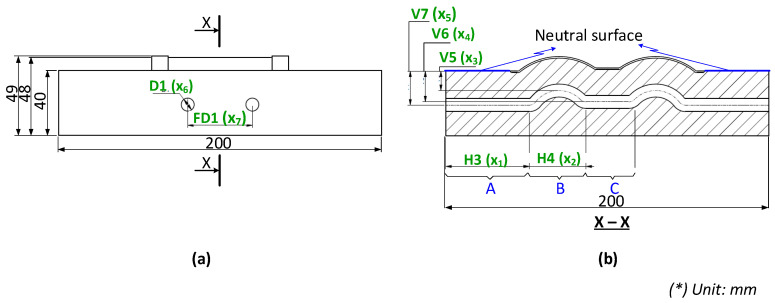
Design parameters for the conformal cooling channel (CCC) on core plate with the side view (**a**) and the cross-section X-X (**b**).

Figure 2 shows the CAD model of a generic injection mold. A proposal for the CCC design parameters has been constructed using the design guidelines shown in Table 1. Figure 3 shows the parameters for the optimization process. Seven parameters must be set to define the shape of the cooling channel fully: channel distance (FD1), diameter (D1), length (H3 and H4), and distance from the centerline of the cooling channel to the mold surface (V7, V6, and V5). The cooling design is shown in Figure 3, and the parameter ranges are defined in Table 2.

### 2.3. Simulation Setup

Hot water was recirculated during the heating process to raise the mold temperature. The water temperature was controlled by a temperature control machine and maintained at 90 °C at the inlet of the cooling channel. At the same time, a manifold collected water at the outlet and recycled it back to the inlet through ducting.

**Figure 4:**
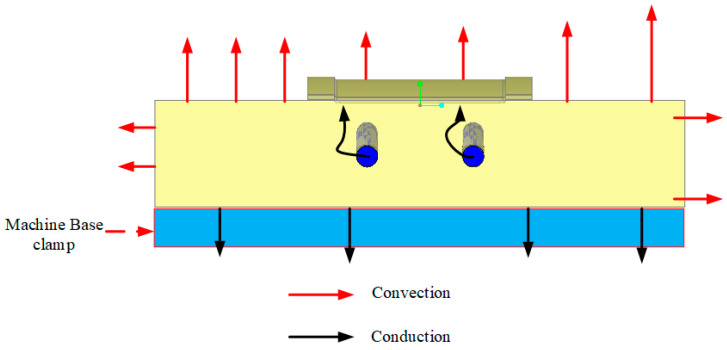
Schematic of heat transfer.

For the mold (solid domain), the material used for the cavity was aluminum alloy. The heat-transfer process included conduction from water in the cooling channel to the mold surface. Heat loss occurred due to heat transfer from the mold surface to the surrounding environment through free convection. The ambient temperature was estimated to be 30 °C. The heat transfer schematic is shown in Figure 4, while the thermal properties of each material are listed in Table 3.

The simulation was based on the experimental setup described. Without loss of generality or oversimplification, the simulation will only focus on the cavity. The simulation used two domains: the solid domain (mold) and two fluid domains (two cooling channels were separated into two domains and used with the same settings). The thermal properties of each material used in the simulation are listed in Table 3 [40], and a schematic of the heat transfer is shown in Figure 4.

**Figure 5:**
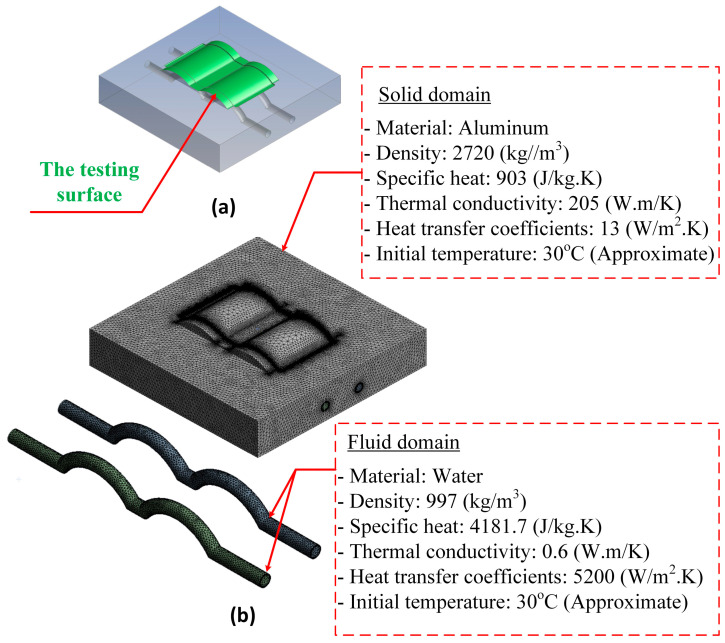
Testing surfaces (**a**) and meshing model (**b**).

As mentioned, the temperature of the cavity surface will directly impact part quality and the filling ability of melted plastic; therefore, this surface is observed and discussed in this study. The temperature was taken on the surface of the mold in contact with the part, shown as a green surface in Figure 5a. The simulation was used to determine the average and differential temperatures on the mold surface. The simulation was performed using the ANSYS software version 19.2 with the meshing model and the boundary conditions, as shown in Figure 5b.

### 2.4. Response Surface Methodology

Response surface methodology (RSM) is an effective method of evaluating the influence of experimental variables on the response. The relationship between the variables and the response is expressed as a surface, also known as the “response surface.” This surface can be represented as a curved surface with two variables in three-way space. However, the response surface becomes a hypersurface in multidimensional space with the addition of experimental variables. This method can advantageously represent the relationship between observed variables and the response through a mathematical model, which can optimize the response and determine individual responses. Parameters include a set of variables to achieve the required value. The mathematical model showing variables and reactions used in this method can be expressed as follows:

(1) $$Y=\beta_{O}+\sum_{i=1}^{k}\beta_{i}\cdot X_{i}+\sum_{i=1}^{k}\beta_{ii}X_{i}^{2}+\sum_{i=1}^{k}\sum_{j=1}^{k}\beta_{ij}\cdot X_{i}\cdot X_{j}+\varepsilon$$

In this equation:

*Y*: response;
$\sum_{i=1}^{k}\beta_{i}\cdot X_{i}$: linear component of the equation;
$\sum_{i=1}^{k}\beta_{ii}X_{i}^{2}$: quadratic component of the equation;
$\sum_{i=1}^{k}\sum_{j=1}^{k}\beta_{ij}\cdot X_{i}\cdot X_{j}$: interaction between variables;
$\beta_{O}$: constant;
ε: error.

These components are not required based on the relation between response and variables. Typically, to correctly determine the equation result, the initial model must fully include these components. A mathematical model was derived using experimental results to find the coefficients in which all components were re-evaluated by the statistical method, and statistically insignificant parts were removed. The mathematical model was built this way until only statistically substantial elements remained.

**Figure 6:**
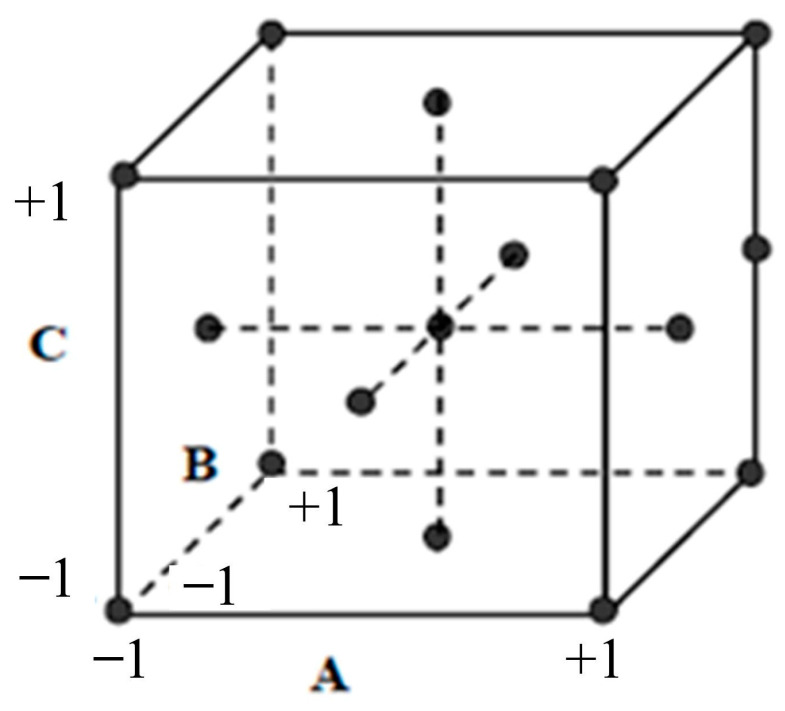
Face–center design schematic.

To find the regression equation coefficients, the figure of variables and responses throughout each experiment was used. Design of experiments (DOE) is an efficient experimental strategy for determining the relationship between variables and responses. The most widely accepted experimental designs are full and fractional factorial, with the number of levels depending on the relationship between variables and responses. A complete factorial design consists of all possible combinations between each level of the variables. Therefore, the number of experiments observed from a complete factorial design is numerous, especially in the case of many variables. In this study, with the number of variables being seven and the number of levels being three, the number of experiments observed from a complete factorial design could be 37 = 2187. Instead of a complete factorial design, a fractional factorial design could be a compelling DOE for this study. There are two primary types of DOE for response surface designs: central composite design (CCD) and Box–Behnken design [48,49,50,51]. The Box–Behnken design has fewer experimental design points than the CCD design and only supports up to three levels of each variable. However, the CCD design has to run more experimental trials than the Box–Behnken. However, the disadvantage of this type of experiment is that some design points are located at the axis, making it impossible to experiment because it is beyond the upper or lower value of the variable. To prevent this, face–center design experiments have been developed. This parameter is a variant of CCD, where α = 1. The schematic of the face-centered design can be derived from Figure 6. In this study, the face–center design has been used to implement the experiments. Each variable was separated into three levels, and the number of experiments was 143.

The experimental data will be imported into Ansys software and simulated to observe the result. In this study, the simulation result and the response differed between the maximum and minimum temperatures (*P*8) and the average temperatures (*P*9). The mathematical model only included significant components at 5% (*p*-value). The regression equation obtained from each response is presented below:

(2) $$P8=88.5-1.559x_{1}-0.767x_{2}-2.356x_{3}+1.239x_{4}-0.197x_{5}+0.775x_{6}-0.699x_{7}+0.076x_{3}^{2}+0.019x_{1}x_{2}\;+0.02x_{1}x_{3}-0.011x_{1}x_{4}+0.022x_{1}x_{5}-0.023x_{3}x_{4}-0.008x_{3}x_{5}+0.004x_{3}x_{7}-0.035x_{4}x_{5}\;+0.005x_{3}x_{7},$$

(3) $$P9=47.04-0.031x_{1}-0.023x_{2}+0.096x_{3}-0.491x_{4}+0.401x_{5}+0.96x_{6}+0.404x_{7}-0.003x_{7}^{2}+0.003x_{1}x_{3}\;+0.007x_{1}x_{4}-0.007x_{1}x_{5}+0.003x_{2}x_{3}+0.003x_{2}x_{4}-0.004x_{2}x_{5}-0.012x_{3}x_{4}-0.02x_{3}x_{5}\;-0.011x_{3}x_{7}+0.013x_{4}x_{5}-0.001x_{4}x_{7}-0.001x_{5}x_{7}+0.019x_{6}x_{7}.$$

The analysis of the variance for all factors with each response is shown in Table 4 and Table 5.

In Equations (2) and (3), all the components, including linear, quadratic, and two-way interaction, appeared in the regression model. Through the analysis of the variance table, the p-value of each element is smaller than 0.05, which means all component appearances on the regression equation have statistical significance. The coefficient of determination (R-sq) also has very high values (98.82% for P8 and 99.80% for P9), proving that the regression equation fits the data. The mathematical model can predict the value of response with each deterministic variable, and the regression equation can also be used to optimize the response value.

### 2.5. Multi-Response Optimization

Optimization with a single response value was relatively easy to find. The optimum value of each response was obtained using numerical methods for regression (2) and (3). However, this study aims to find the optimum shape of cooling; with this cooling shape, the temperature could be distributed uniformly on the mold surface. If only one response has been used to find the optimum cooling form, this shape may bring the highest temperature on the mold surface, but the different temperatures may be large and contrary. These numerical results can be seen in Table 6. These two observed simulation results showed that with a higher average, the temperature difference increased. The primary purpose of optimization was to find the best value of each variable such that the average temperature is highest, and the contrast of temperature is minimized.

Solving this issue, the desirability function approach could be used. This function was developed by Harrington and his partner [52] and then popularized by Derringer and Suich [53]. The main idea of this approach is to transform the response function into a suitable function and then optimize it. The overall desirability function was synthesized from the expectation functions of each response [54,55,56]. The object here maximizes the overall desirability function. The overall desirability function can be determined by the function below:

(4) $$D=\sqrt[{k_{1}+k_{2}+\ldots +k_{i}}]{d_{1}^{k_{1}}\cdot d_{2}^{k_{2}}\cdot \ldots \cdot d_{i}^{k_{i}}}$$

In this function:

*d_i_*: desirability function of response *i*;
*k_i_*: the importance coefficient of response *i*.

The desirability of each response received values from 0 to 1. The desirability *d_i_* of response I could be presented as the function below:

When the optimum object is at the maximum:

(5) $$d_{i}=\left\{\begin{array}{c} 0\;khiy_{i}<L_{i}\\ {\left(\frac{y_{i}\;-\;L_{i}}{T_{i}\;-\;L_{i}}\right)}^{w_{i}}\;khi{L_{i}<y}_{i}<T_{i}\\ 1\;khiy_{i}\geq L_{i}. \end{array}\right.$$

When the optimum object is at the minimum:

(6) $$d_{i}=\left\{\begin{array}{c} 1\;khiy_{i}<T_{i}\\ {\left(\frac{U_{i}\;-\;y_{i}}{U_{i}\;-\;T_{i}}\right)}^{w_{i}}\;khi{T_{i}<y}_{i}<U_{i}\\ 0\;khiy_{i}\geq U_{i}. \end{array}\right.$$

In this formula:

*L_i_*: the upper bound value;
*U_i_*: the lower bound value;
*T_i_*: the desirability value;
*w_i_*: weight of each response.

**Figure 7:**
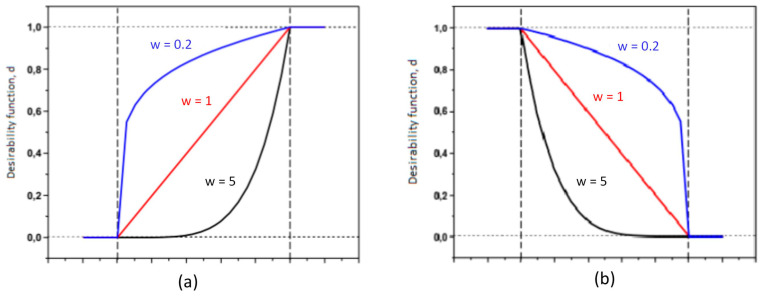
Desirability function of the maximum optimization (**a**) and the minimum optimization (**b**).

The value of the weight when describing the expected function will determine the shape of the graph representing this function. When we choose a weight larger than 1, we emphasize the importance of attaining an objective function close to the desired value. Conversely, when the weight is less than 1, we extend the acceptance more when the objective function is far from the desired value. Figure 7 shows the desirability of the maximum and minimum optimization when the weight of each response is 0.2, 1, and 5.

**Figure 8:**
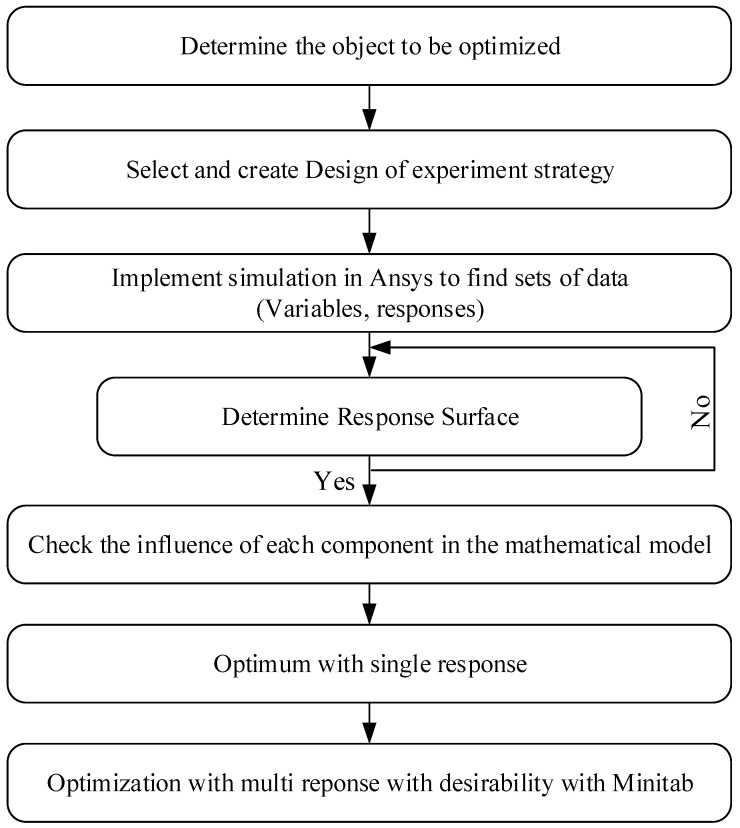
Optimization process diagram.

In this study, the P8 (temperature difference) optimized object was depreciation, and the P9 (average temperature) optimized object was maximization. The optimization process is illustrated in the figure. A regression equation based on the CCD method was used to find the optimum point. The optimization process with the desirability function approach could be easily solved using an added tool such as Minitab, and the progress is shown in Figure 8.

**Figure 9:**
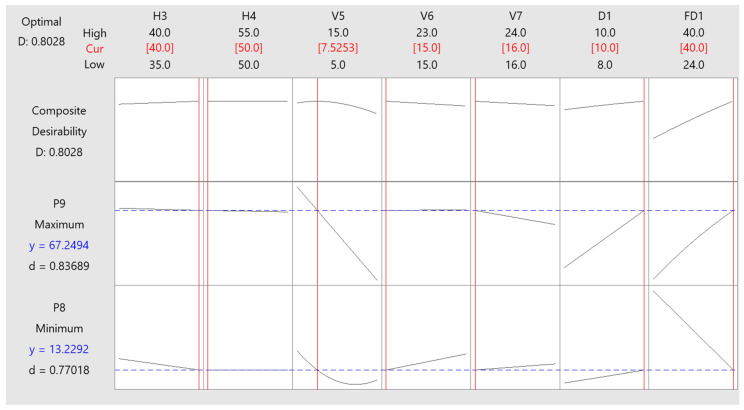
Optimization plots for responses P8 and P9.

The optimization plots for each response are shown in Figure 9. The optimum point and predicted value of each response were determined.

The result in Table 7 shows that the optimized value of each response was 13.2292 °C, which is far from the target value (8.5874 °C), so the desirable value was relatively low (77.02%), but it is still in the acceptable range (8.5874 °C ≤ P8 ≤ 28.7853 °C). On the contrary, the average temperature response optimized value was predicted at 67.2494 °C, close to the target value (69.5697 °C); therefore, the desirable value was relatively high (83.69%). The overall desirable value for these two responses was 80.28% (composite desirability); this value could be accepted.

**Figure 10:**
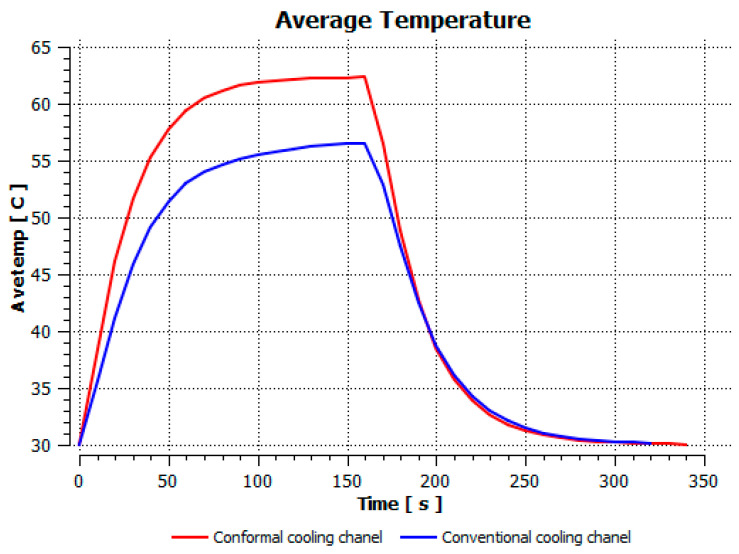
Average temperature between the CCC and a conventional cooling channel.

The optimum point of each variable obtained from the optimization process was used to modify the shape of the cooling channel and rerun the simulation on Ansys software to verify the values predicted. In addition to the heating process, the simulation analyzed the cooling process. In the cooling process, water at 30 °C was used. A comparison between the prediction and simulation values of the heating process is shown in Table 8. It can be seen that the difference between the prediction value and simulation value is minimal (0.79% with P9 response and 4.8% with P8 response). Figure 10 shows the average temperature history of the testing surface when the CCC and the conventional channel were used. This result shows that the CCC supports the higher heat transfer coefficient for the heating period, which could keep a higher temperature at the end of the heating step. The temperature distribution at the core surface could be observed at the end of the heating step (Table 9) and the end of the cooling phase (Table 10). In the heating process, the average temperature surged quickly in the first 60 s, and it was consistently more significant in the mold that used a CCC than the mold that used a conventional cooling channel; in the steady state, the average temperature on the CCC mold was 66.72 °C, while the traditional cooling channel only reached 63.08 °C. It can be seen from Table 9 that in the CCC mold, the high temperature (>71 °C) was distributed over a large area on the mold surface. Still, in the conventional cooling channel mold, the heat zone with the most significant size had a temperature of only 67 °C. Meanwhile, in the simulation, the cooling process used water at 30 °C on both types of molds. The figure in Table 10 clearly shows the effectiveness of each cooling channel type. The temperature distribution of the mold using the CCC was more uniform than the mold that used the straight cooling channel.

Table 11 shows the temperature distribution at each mold section compared to the mold used in CCC, which used a straight cooling channel. In the two types of molds, it is evident that the temperature decreases when moving away from the surface where there is contact with the high-temperature fluid to the top surface of the mold, but the figure shows a big difference between the two types of molds. In sections A-A and B-B, the temperature decreased from approximately 77 °C to 72 °C at the top surface. The temperature dropped by 5 °C in the mold using a CCC. Meanwhile, in the mold that used a straight cooling channel, the temperature was reduced by 9.8 °C from the maximum temperature at contact with the hot water surface (75.5 °C) to the temperature at the top surface (65.68 °C). In sections C-C and D-D within the mold that used the CCC, the temperature decreased by 4.9 °C in the vertical axis from the cooling channel to the top surface, and this decreased from 72.07 °C to 64.45 °C when moving from the cooling channel’s centerline to the boundary edge of the top surface, and to 71.4 °C in the middle of the mold. The amount of temperature relieved was 6.6 °C in the horizontal side. In the same section, the temperature decreased significantly more in the mold that used a straight cooling channel than in the mold that used CCC on both sides, vertical and horizontal. More specifically, when moved from the cooling channel surface to the top surface, the temperature went down 8.1 °C, which means double that of the CCC mold, from 74.9 °C to 66.8 °C. Along the horizontal side, the temperature decreased by 6.9 °C from the middle of the mold to the boundary edge.

### 2.6. Experimental Verification

**Figure 11:**
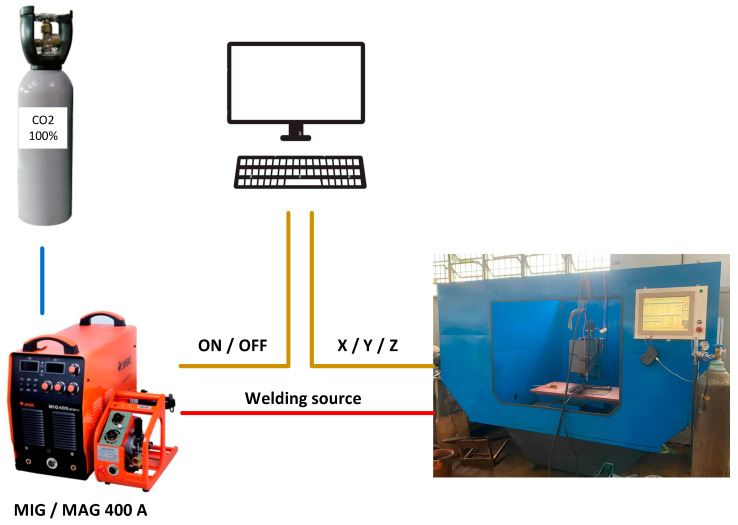
Manufacturing equipment for the GMAW deposition and milling process.

Two models were manufactured to verify the simulation results for two types of cooling channels (CCC and straight cooling channel). The fabrication of the cavity mold with a linear cooling channel was simple, but the process was more difficult with the CCC mold. In this research, the mold plate with two CCC was manufactured by the WAAD combined with the milling method. The proposed MAG welding system introduces a metal deposition process via gas metal arc welding (GMAW) to manufacture a mold plate featuring two conformal cooling channels. Essentially an additive manufacturing (AM) method, it involves the deposition of a weld bead in a track-by-track and layer-by-layer manner. To facilitate this, a machine setup was constructed by affixing a GMAW torch to a computer numerical control (CNC) machine, as depicted in Figure 11. An ER70S-6 wire (diameter 1.2 mm) was used in this setup. In order to minimize weld splash, an optimal set of process parameters were determined: voltage of 19.5 V, current of 115 A, standoff distance of 8.5 mm, shielding gas flow rate of 18 L/min, and a travel speed of 250 mm/min. Subsequent to the additive manufacturing, the milling method was utilized to polish the surface and create a precise, finished product.

**Figure 12:**
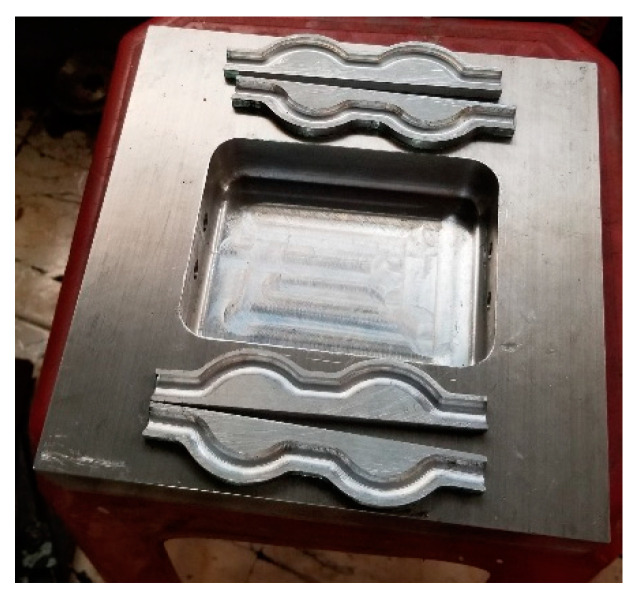
Insert for forming the CCC in the WAAD process.

**Figure 13:**
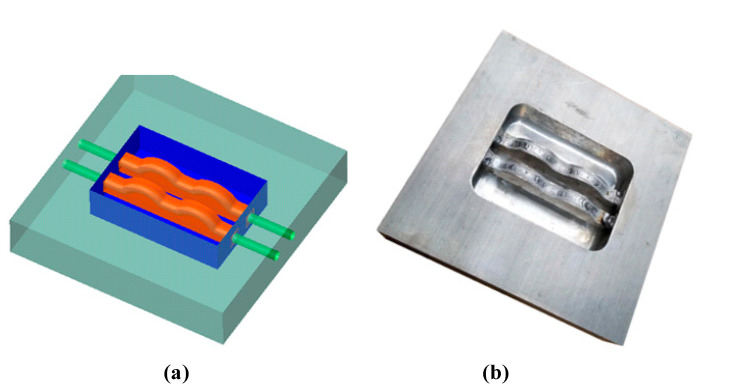
Cooling channel assembled to the mold base with the 3D model (**a**) and the experimental part (**b**).

**Figure 14:**
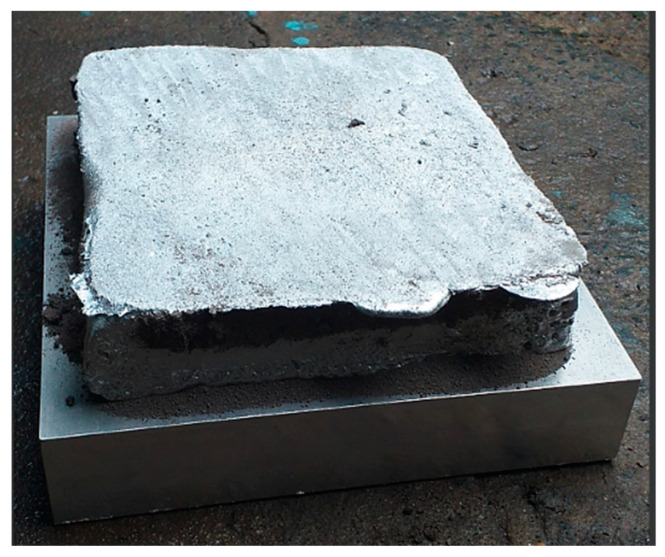
Mold base generated with WAAM.

**Figure 15:**
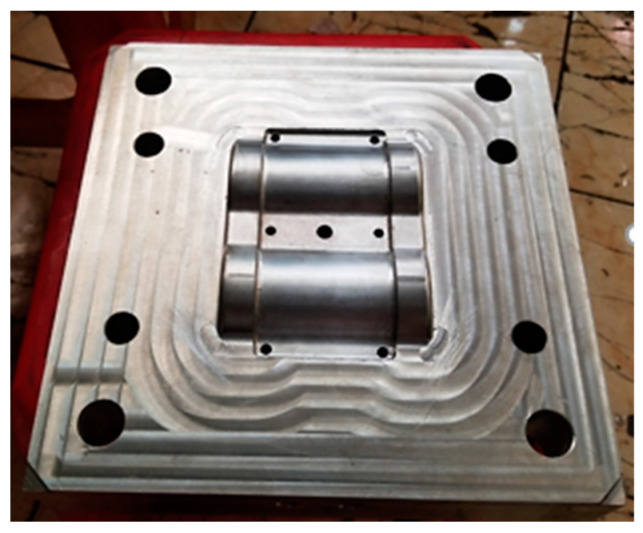
Finished model.

For the creation of the conformal cooling channel (CCC), two inserts were pre-manufactured, each one possessing a profile that matched the optimized parameters (as seen in Figure 12). These inserts were then welded together as displayed in Figure 12. The assembled inserts were then fitted into the plate via a strategically placed hole (Figure 13). The WAAM process was then carried out to deposit the additive material, with the results visible in Figure 14. At the conclusion of the WAAM process, it was observed that the plate’s geometry did not perfectly align with the intended design. However, this issue was resolved through the milling process, with the final plate displayed in Figure 15.

This finalized plate was then integrated with the other mold components in preparation for molding, as well as for the observation of temperature distribution across the mold during the molding process.

**Figure 16:**
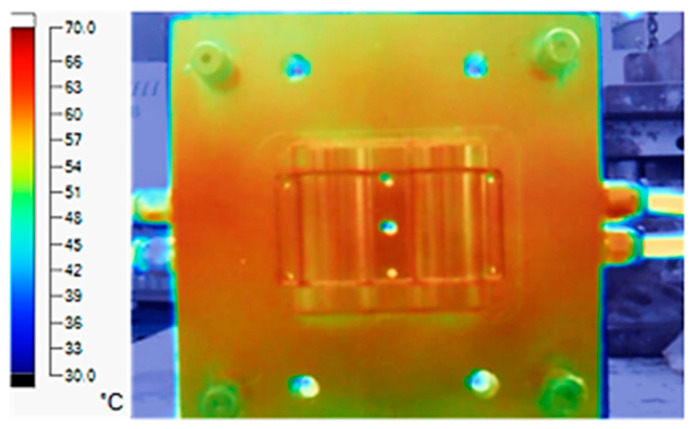
Straight cooling channel heating result.

**Figure 17:**
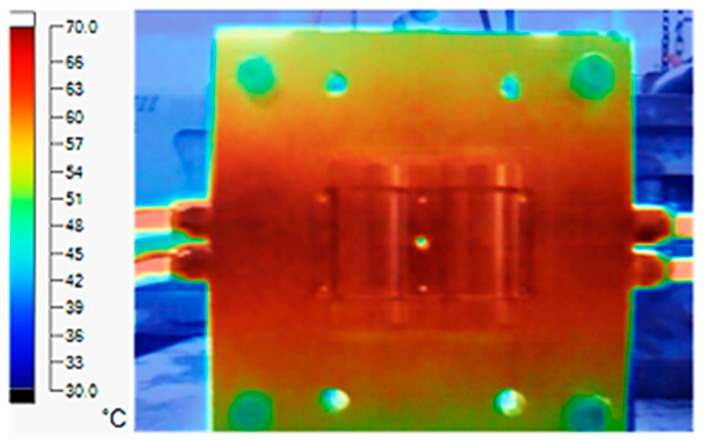
CCC heating.

**Figure 18:**
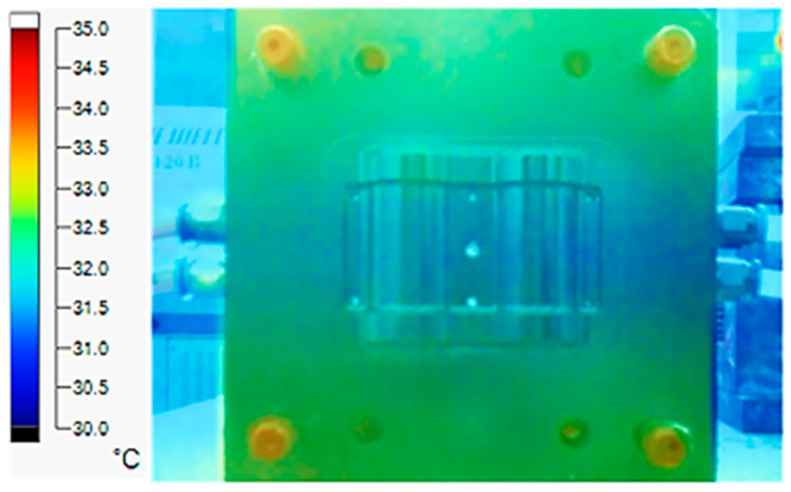
Straight cooling channel cooling result.

**Figure 19:**
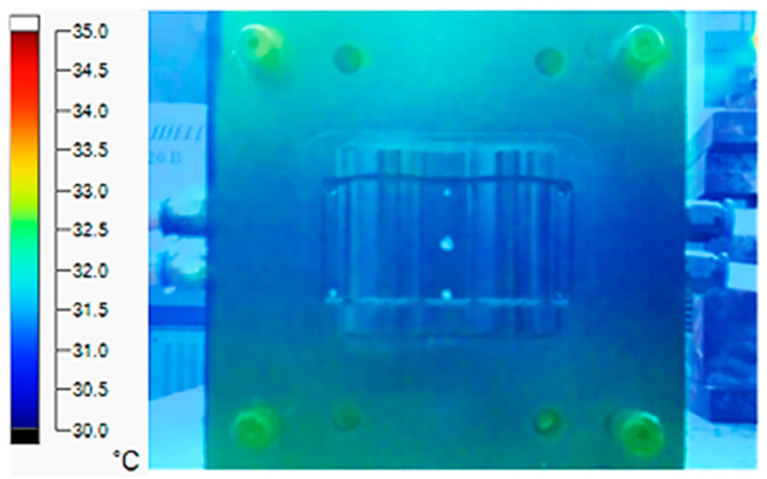
CCC cooling result.

The experiment was implemented using hot water at 80 °C. An infrared camera was used to capture the temperature of the mold surface at the end of the heating and cooling steps. The temperature distributions on the mold surface at the end of the heating and cooling steps are shown in Figure 16, Figure 17, Figure 18 and Figure 19.

During the heating step, Figure 16 and Figure 17 show that when comparing the simulation and experimental results, the deviation between these two results does not vary much. Specifically, within the straight cooling channel mold, the simulation results for the average and maximum temperature were 63.01 °C and 66.8 °C, respectively, whereas the experimental results for those parameters were 64 °C and 67.8 °C. With the CCC mold, the simulation result nearly matched the experimental results. In the simulation, the average and maximum temperatures were 66.72 °C and 72.07 °C, respectively, and the experiment yielded 64.6 °C and 68.4 °C, respectively. The deviation between the simulation and the investigation was insignificant. The straight cooling channel mold was from 1 °C to 3.3 °C, whereas the CCC mold temperature deviation was from 2.12 °C to 4 °C. This error can be readily accepted because of the differences between experimental and simulation conditions, such as environmental effects, machining-modeling errors, and mold material variations. In addition, the material properties do not perfectly conform to the ideal material used in the simulation, and all these factors are attributed to the observed variations.

When comparing the experimental results between the straight and CCCs, the latter displayed more heating and cooling effectiveness on the heating mold. The temperature distribution of the CCC mold surface was more uniform than that of the straight cooling channel. The average temperature of the CCC mold was higher than the straight cooling channel mold (64.6 °C and 63.01 °C, respectively). Throughout the experiment, the CCC mold temperature remained higher than that of the straight cooling channel.

The same conditions were used for the cooling process, and the results were similar to the simulation. First, looking at the experimental result in Figure 18, the straight cooling channel mold surface shows a maximum temperature of approximately 34 °C. However, as shown in Figure 19, the temperature distribution of the mold surface was more uniform at about 31 °C. There was no point on the surface of the CCC mold where the temperature was higher than 34 °C.

**Figure 20:**
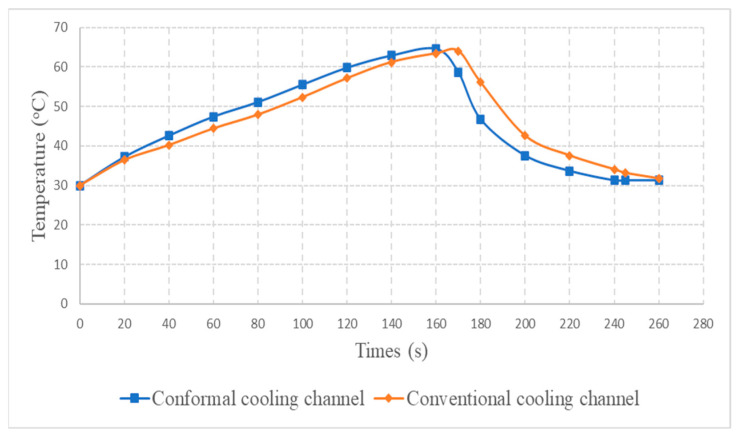
Temperature history of the testing surface for the CCC and conventional cooling channels.

Figure 20 shows the temperature distribution of the mold using a conformal and straight cooling channel during the experiment. The mold using a CCC had a faster heating speed than the other mold using a linear channel. Specifically, as shown in the figure, during the heating process, the brown lines (heating of the CCC) were always above the blue bars (heating of the straight channel), indicating that at any given time the cavity temperature of the CCC mold was higher than that of the linear channel. In the cooling process, the brown lines were always below and had a steeper slope than the blue lines, demonstrating that the 3D channel cooling capacity was higher than that of the straight channel. The temperature distribution graphs also show that the mold using a CCC had faster heating and cooling rates.

The simulation and experimental results could be compared, as presented in Figure 10 and Figure 20. The results show that this deviation is relatively small, and the results can be considered satisfactory.

## 3. Conclusions

This study determined the effectiveness of cooling channel geometry on the heat transfer process using the response surface methodology of two regression equations, which were modeled for average and varying temperatures with upper and lower bounds. The optimum geometry for the cooling channel was defined using these two equations. The effectiveness of the CCCs was demonstrated through analysis and verified experimentally. The average temperature was consistently higher when the CCC heated the mold. Additionally, the high-temperature heat zone in a mold that used a CCC was more significant than in molds using conventional cooling channels.

## Figures and Tables

**Table 1:** Design guidelines for cooling channels.

Product Thickness	Channel Diameter	Distance between Two Channels	Distance from the Channel Centerline to the Mold Surface
2 mm	8–10 mm	24~40 mm	16~30 mm
4 mm	10–12 mm	30~48 mm	20~36 mm
6 mm	12–15 mm	36~60 mm	24~45 mm

**Table 2:** CCC design parameter range.

	Lower Bound (mm)	Upper Bound (mm)
H3 (x_1_)	35	40
H4 (x_2_)	50	55
V5 (x_3_)	5	15
V6 (x_4_)	15	23
V7 (x_5_)	16	24
D1 (x_6_)	8	10
FD1 (x_7_)	24	40

**Table 3:** Density and thermal properties of aluminum and water.

	Density (kg·m^−3^)	Specific Heat (J·kg^−1^·K^−1^)	Thermal Conductivity (W·m^−1^·K)	Heat Transfer Coefficients (W·m^−2^·K)
Water	997.0	4181.7	0.6	5200
Aluminum	2720	903	205.00	13

**Table 4:** Analysis of variance for all factors with response P8.

Source	DF	Adj SS	Adj MS	F-Value	*p*-Value
Model	17	3941.18	231.83	617.16	0.000
Linear	7	3802	543.14	1445.89	0.000
H3	1	13.84	13.84	36.84	0.000
H4	1	2.12	2.12	5.64	0.019
V5	1	1018.71	1018.71	2711.9	0.000
V6	1	2.08	2.08	5.55	0.020
V7	1	32.54	32.54	86.62	0.000
D1	1	77.98	77.98	207.59	0.000
FD1	1	2654.73	2654.73	7067.12	0.000
Square	1	42.39	42.39	112.84	0.000
V5*V5	1	42.39	42.39	112.84	0.000
Two-Way Interaction	9	96.79	10.75	28.63	0.000
H3*H4	1	1.82	1.82	4.85	0.030
H3*V5	1	8.08	8.08	21.51	0.000
H3*V6	1	1.62	1.62	4.3	0.040
H3*V7	1	6.18	6.18	16.44	0.000
V5*V6	1	28.2	28.2	75.07	0.000
V5*V7	1	3.51	3.51	9.35	0.003
V5*FD1	1	3.86	3.86	10.29	0.002
V6*V7	1	40.57	40.57	108	0.000
V6*FD1	1	2.96	2.96	7.88	0.006
Error	125	46.96	0.38		
Total	142	3988.14			
S	R-sq		R-sq (adj)		
0.612889	98.82%		98.66%		

**Table 5:** Analysis of variance for all factors with response P9.

Source	DF	Adj SS	Adj MS	F-Value	*p*-Value
Model	21	1889.03	89.95	2861.98	0.000
Linear	7	1824.79	260.68	8293.95	0.000
H3	1	0.43	0.43	13.64	0.000
H4	1	0.15	0.15	4.67	0.033
V5	1	1151.52	1151.52	36,636.8	0.000
V6	1	1.32	1.32	42.13	0.000
V7	1	16.31	16.31	519.04	0.000
D1	1	313.69	313.69	9980.47	0.000
FD1	1	341.37	341.37	10,860.9	0.000
Square	1	0.57	0.57	18.04	0.000
FD1*FD1	1	0.57	0.57	18.04	0.000
Two-Way Interaction	13	63.67	4.9	155.83	0.000
H3*V5	1	0.18	0.18	5.74	0.018
H3*V6	1	0.57	0.57	18	0.000
H3*V7	1	0.71	0.71	22.45	0.000
H4*V5	1	0.24	0.24	7.53	0.007
H4*V6	1	0.13	0.13	4.02	0.047
H4*V7	1	0.23	0.23	7.21	0.008
V5*V6	1	7.25	7.25	230.55	0.000
V5*V7	1	20.88	20.88	664.29	0.000
V5*FD1	1	25.12	25.12	799.21	0.000
V6*V7	1	5.31	5.31	168.9	0.000
V6*FD1	1	0.14	0.14	4.47	0.036
V7*FD1	1	0.12	0.12	3.73	0.056
D1*FD1	1	2.82	2.82	89.74	0.000
Error	121	3.8	0.03		
Total	142	1892.83			
S	R-sq		R-sq (adj)		
0.177287	99.80%		99.76%		

**Table 6:** Simulation result of two combination variables.

H3	H4	V5	V6	V7	D1	FD1	P8	P9
35	50	5	23	24	8	24	13.08	62.94
35	50	5	23	16	8	40	10.03	62.64

**Table 7:** Optimum point and predicted values of each response.

Solution	H3	H4	V5	V6	V7	D1	FD1	P9 Fit	P8 Fit	Composite Desirability
1	40	50	7.53	15	16	10	40	67.2494	13.2292	0.802845

**Table 8:** Comparison between RSM prediction and simulation results.

	H3	H4	V5	V6	V7	D1	FD1	P9	P8
Prediction	40	50	7.5	15	16	10	40	67.25 °C	13.23 °C
Simulation								62.34 °C	12.59 °C

**Table 9:** Temperature distribution of the testing surface during the heating process.

	Conformal Cooling Channel	Conventional Cooling Channel
Heating process	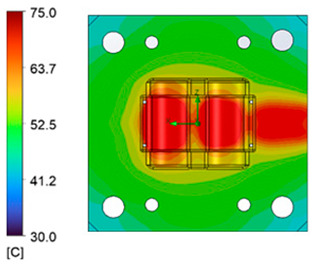	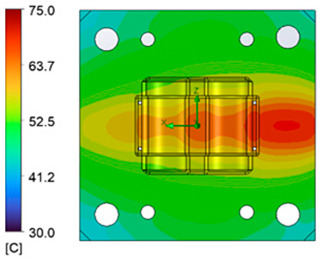

**Table 10:** Temperature distribution of the mold surface during the cooling process.

	Conformal Cooling Channel	Conventional Cooling Channel
Cooling process	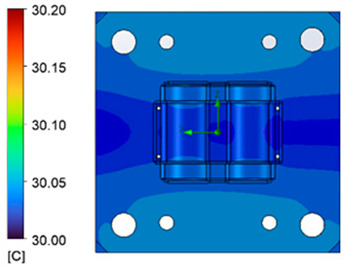	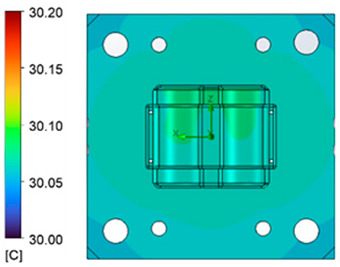

**Table 11:** Temperature distribution at each section.

	Conformal Cooling Channel	Conventional Cooling Channel
	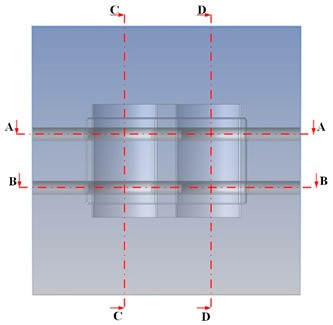	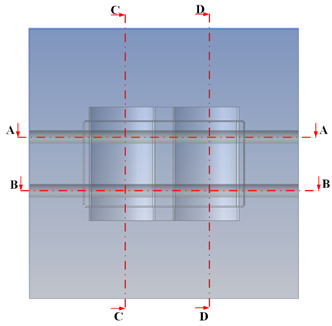
A-A	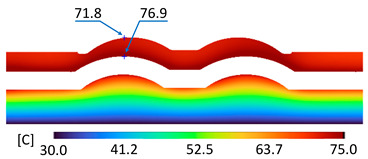	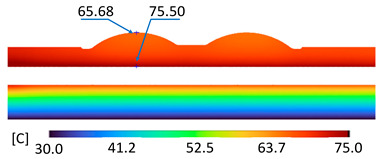
B-B	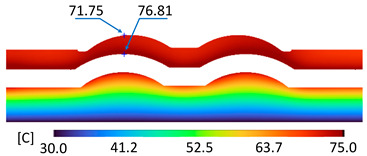	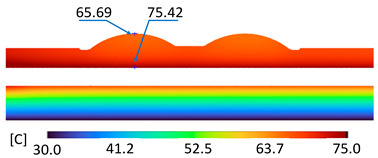
C-C	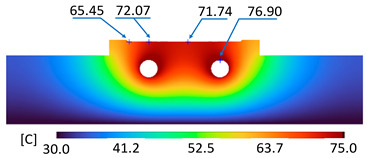	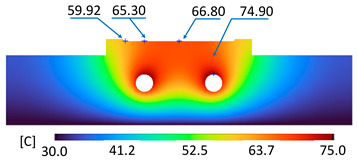
D-D	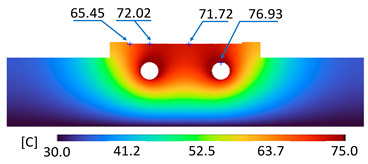	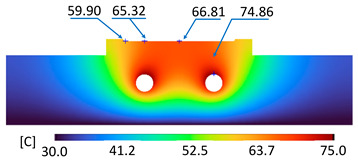

## Data Availability

The data used to support the findings of this study are available from the corresponding author upon request.
